# Light-sensitive brain pathways and aging

**DOI:** 10.1186/s40101-016-0091-9

**Published:** 2016-03-15

**Authors:** V. Daneault, M. Dumont, É. Massé, G. Vandewalle, J. Carrier

**Affiliations:** 1 Functional Neuroimaging Unit, University of Montreal Geriatric Institute, Montreal, QC Canada; 2 Center for Advanced Research in Sleep Medicine, Hôpital du Sacré-Cœur de Montréal, Montreal, QC Canada; 3 Department of Psychology, University of Montreal, Montreal, QC Canada; 4 Cyclotron Research Centre, University of Liège, Liège, Belgium

**Keywords:** Light, Aging, Brain, Non-image-forming (NIF) functions

## Abstract

Notwithstanding its effects on the classical visual system allowing image formation, light acts upon several non-image-forming (NIF) functions including body temperature, hormonal secretions, sleep-wake cycle, alertness, and cognitive performance. Studies have shown that NIF functions are maximally sensitive to blue wavelengths (460–480 nm), in comparison to longer light wavelengths. Higher blue light sensitivity has been reported for melatonin suppression, pupillary constriction, vigilance, and performance improvement but also for modulation of cognitive brain functions. Studies investigating acute stimulating effects of light on brain activity during the execution of cognitive tasks have suggested that brain activations progress from subcortical regions involved in alertness, such as the thalamus, the hypothalamus, and the brainstem, before reaching cortical regions associated with the ongoing task. In the course of aging, lower blue light sensitivity of some NIF functions has been reported. Here, we first describe neural pathways underlying effects of light on NIF functions and we discuss eye and cerebral mechanisms associated with aging which may affect NIF light sensitivity. Thereafter, we report results of investigations on pupillary constriction and cognitive brain sensitivity to light in the course of aging. Whereas the impact of light on cognitive brain responses appears to decrease substantially, pupillary constriction seems to remain more intact over the lifespan. Altogether, these results demonstrate that aging research should take into account the diversity of the pathways underlying the effects of light on specific NIF functions which may explain their differences in light sensitivity.

## Background

### Two functional systems detecting light: photoreceptor contribution and neural pathways

From a functional point of view, there are two systems detecting light in mammals and humans. The first one is the classical visual system responsible for image formation, and the second one is the non-image-forming (NIF) system which detects environmental irradiance and contributes to modulation of many fundamental functions in living organisms. The physiological, behavioral, and cognitive functions which are modulated by light but not associated with conscious image perception are called NIF functions. These responses include circadian entrainment and shift the timing of circadian rhythms such as hormone secretion (melatonin, cortisol), heart rate, body temperature, and the sleep-wake cycle. These NIF effects are detected hours or days following light exposure. NIF responses also include acute physiological effects of light detected more rapidly, including melatonin suppression, pupillary constriction, alertness, and performance improvement as well as cognitive brain responses [1–5].

### Melanopsin retinal ganglion cells

In the course of the year 2000s, the discovery of melanopsin (OPN4)-photosensitive pigment expressed by intrinsically photosensitive retinal ganglion cells (ipRGC) contributed to a better understanding of the neural bases of the NIF system [6]. The crucial importance of OPN4 in NIF responses has been corroborated by animal and human studies [7–10]. In humans, melanopsin is expressed in a small subset of cells representing only 1–2 % of all retinal ganglion cells (RGC) [1, 10–14]. These photoreceptors measure the intensity of light (irradiance detection) with a maximum sensitivity toward short light wavelength (blue ~ 460–480 nm) [6, 7, 11]. Melanopsin ipRGC have a low spatial resolution and long latencies as compared to cone and rod responses, and they show the ability to integrate photic energy over long periods of time [6, 7, 13, 14]. To date, five ipRGC subtypes (M1–M5) have been identified according to morphological, molecular, and functional characteristics [8, 11, 15]. M1 have more melanopsin pigment than all other subtypes, and they can be subdivided according to the transcription factor Brn3b (Brn3b positive-M1 versus Brn3b-negative M1) [16–18]. M2 have extended dendrites and soma. M2 also shows more complex connections than M1 including afferents from the rods and cones suggesting that their intrinsic photic response might be more modulated by inputs from classical photoreceptors [18]. M3 has similar characteristics to M2, with intermediate levels of melanopsin [15, 19] and M4–M5 possess long dendrites, abundant arborization, and very low levels of melanopsin (i.e., low intrinsic light response) [15, 18–23]. M1 to M5 project to specific subcortical brain areas and play different functional roles in the NIF and in the classical visual systems [16, 22].

### Visual and non-visual neural pathways

#### Classical visual system: image forming system

**Fig. 1:**
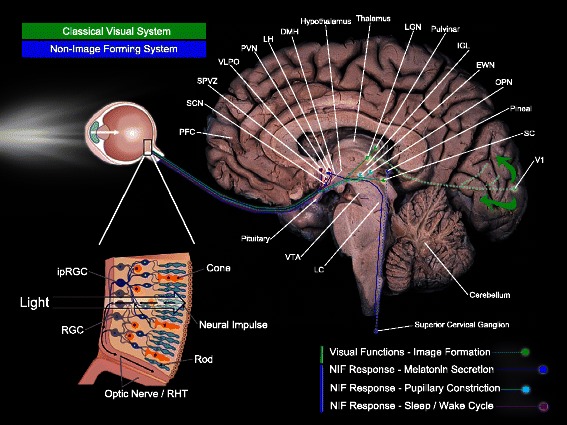
Light-sensitive brain pathways. Simplified brain networks (not exhaustive representation) of the classical visual system and the non-image-forming system. Abbreviations: *PFC* prefrontal cortex, *SCN* suprachiasmatic nucleus, *SPVZ* subparaventricular zone, *VLPO* ventrolateral preoptic nucleus, *PVN* paraventricular nucleus of the hypothalamus, *LH* lateral hypothalamus, *DMH* dorsomedial nucleus of the hypothalamus, *LGN* lateral geniculate nucleus, *IGL* intergeniculate leaflet, *EWN* Edinger-Westphal nucleus, *OPN* olivary pretectal nucleus, *SC* superior colliculus, *V1* primary visual area, *LC* locus coeruleus, *VTA* ventral tegmental area, *ipRGC* intrinsically photosensitive retinal ganglion cell, *RHT* retino-hypothalamic tract. Eye illustration components modified from: http://2012books.lardbucket.org/books/beginning-psychology/s08-02-seeing.html —reproduction/modifications in accordance with: http://creativecommons.org/licenses/by-nc-sa/3.0/ Brain template: reproduced with permission from McGraw-Hill Education Material (source: Saladin, Kenneth S., Human Anatomy, Edition: 2, ISBN: 9780072943689, Figure 15.2-b, p. 425)

Specific neural pathways are described for visual and non-visual systems (Fig. 1). Beginning with the eye, the classical visual system uses mainly rods and cones for image formation but also ipRGC for rudimentary visual functions [20, 22]. Cones are responsible for photopic vision (higher light intensity) with high spatial acuity and color discrimination. The classical photopic system in humans includes three types of cones showing mean peak sensitivity (*λ*_max_) at 555 nanometers (nm), i.e., the green part of the light visible spectrum. S-cones express the short-wavelength-sensitive opsin cyanolabe (*λ*_max_ 420 nm), M-cones express chlorolabe opsin (*λ*_max_ 535 nm), and L-cones express a red-shifted opsin, the erythrolabe (*λ*_max_ 565 nm) [24]. Scotopic vision (i.e., contrast detection, dim light vision) is sustained by rods [25] using rhodopsin photopigment (*λ*_max_ 507 nm in humans) [24]. Using the optic tract, the brain pathways of the classical visual system project to subcortical nucleus, such as the thalamic lateral geniculate nucleus (LGN), the superior colliculus (SC), and the lateral posterior pulvinar complex (Pul-LP), before reaching the primary visual occipital area (V1) and then at other neocortical regions engaged in dorsal and ventral visual attentional brain pathways [26–29] (Fig. 1). Animal studies show that ipRGC (possibly non-M1 subtypes [22, 23]) also send projections to dorsal LGN (dLGN) and SC [16, 17, 22, 23, 30, 31]. These ipRGC projections play a role in conscious perception of spatial brightness and speed motion [16, 31–33]. Recent animal evidences also support the functional role of melanopsin-expressing ipRGC projections to dLGN in visual responses optimization with irradiance detection [33]. Overall, complex interactions between classical (cones, rods) and non-classical (melanopsin-expressing ipRGC) photoreceptors and their projections contribute to the classical visual system [16, 17, 20, 32].

#### Non-visual system/non-image-forming system

The second system, namely, the NIF system, uses ipRGC in addition to rods and cones and shows a peak sensitivity in the blue part of the light spectrum (~460–480 nm) [6, 7, 11, 13, 14, 31, 34]. A monosynaptic pathway, the retinohypothalamic tract (RHT), conveys light information from ipRGC axons [35, 36]. As illustrated in Fig. 1, the NIF system directly projects via the RHT to subcortical regions engaged in melatonin secretion, pupillary constriction, and the regulation of the sleep-wake cycle [2, 37, 38].

RHT directly connects the ipRGC from the retina to the suprachiasmatic nuclei (SCN) of the anterior hypothalamus, the master circadian oscillator (biological clock) [1, 11, 39]. SCN is the endogenous master biological clock that allows temporal organization of living organisms, synchronizing circadian rhythms among themselves as well as with the external environment. SCN sends efferent projections to the hypothalamic and non-hypothalamic structures [30], including the paraventricular nucleus of the hypothalamus (PVN), the dorsomedial nucleus of the hypothalamus (DMH), and finally, the intergeniculate leaflet (IGL) of the thalamus which also sends projections to SCN [40]. Interactions between the SCN, the PVN, the superior cervical ganglion (SCG), and the pineal gland support the neural network of melatonin suppression [41] (see Fig. 1 melatonin suppression). Without being exhaustive here, many brain areas other than the SCN also receive direct projections from the ipRGC. Thus, olivary pretectal nucleus (OPN), the crucial node of the pupillary constriction pathway, receives direct projections from the ipRGC. OPN sends projections to the Edinger-Westphal nucleus (EWN) which in turn, innervate the sphincter muscle of the pupil allowing pupillary constriction [42]. The ipRGC also sends direct connections to regions engaged in the regulation of the sleep-wake cycle [2, 37, 38], such as the ventrolateral preoptic nucleus (VLPO; sleep-wake regulation core-region), the subparaventricular nucleus/zone (SPVZ) of the hypothalamus, which is involved in sleep regulation but also in motor activity, as well as the lateral hypothalamus (LH), which contains orexin (hypocretin) neurons regulating wakefulness [20, 22, 30, 40]. Furthermore, light may also affect the sleep-wake cycle via the connections between the SCN and the DMH since the DMH also sends projections to the VLPO, the LH, and the locus coeruleus (LC) [40, 43, 44]. The amygdala, a structure involved in emotional processes, also receives direct projections from the ipRGC [30, 31] and might represent a key target of the NIF system by potentiating effects of light on alertness and mood. This limbic area is part of the neural network named the “Salience Network” associated with responsiveness to stimuli [45].

#### Photoreceptor contribution to NIF responses

Light stimulus characteristics influence the photoreceptor’s contribution to specific NIF responses. For instance, light intensity, wavelength, and temporal characteristics define the specific photoreceptor’s contribution to pupil light reflex (PLR) [46–49]. At low light intensities, rods and cones contribute to PLR but cones’ contribution decreases as the duration of light exposure increases and is minimal beyond 30 s [47, 48]. At high light intensities (>12 log units per ph/cm^2^/s), ipRGC mainly contributes to the sustained PLR [8, 47, 50], i.e., in response to light exposure extending beyond 30 s.

Recently, complex photoreceptor interventions/communications have also been reported for circadian entrainment. Blue-yellow cone’s color discrimination/opponency seem to modulate the ipRGC signal transmission to SCN neurons making them sensitive to color [51]. Thus, SCN cells would be sensitive to both brightness and color. This could correspond to an evolutionary strategy using color as a time-of-day indicator based on spectral composition of the solar cycle and twilight transition [51].

Studies have reported that 80 % of all ipRGC projections to the SCN are from M1 Brn3b-negative and 20 % are from M2 [21, 52]. In contrast, 45 % of ipRGC projections to the OPN (pupillary constriction) are from M1 Brn3b-positive (shell part) and 55 % are from M2 subtype (core part) [10, 21, 52]. Relative contribution of each photoreceptor and interactions still need to be determined for specific NIF functions [2, 10, 23]. The classical visual system and the NIF system are different by their respective functions but evidences now reveal that a complete dichotomy of these two systems is outdated at the eye and brain levels. An integrative hypothesis suggesting a multi-dimensional system with a relative segregation of different networks, rather than their full independence, seems more likely based on the observed data. Further research will help identify retinal and neural networks involved in the effects of light for each NIF functions.

Overall, as for the classical visual pathway, the underlying neural pathways of the NIF system are complex and several brain areas are involved in the mechanisms by which changes in the quality of the light environment affect various NIF functions [22, 30, 31].

### Effects of light on alertness and cognitive functions: short versus longer wavelengths

In agreement with the peak sensitivity of each light-detecting system, many studies have confirmed greater sensitivity of non-visual responses under blue monochromatic light exposure (~460–480 nm), in comparison to longer wavelengths such as green monochromatic light [4, 5, 53–59]. Hence, the impact of light on sleepiness, alertness, performance, as well as the modulation of cognitive brain functions are greater under blue monochromatic light and blue-enriched light exposure, as compared to longer light wavelengths [4, 5, 53–57, 60]. Lower levels of subjective sleepiness [53, 61, 62], but also of objective alertness as measured with electroencephalogram (EEG), are reported under blue light exposure, as compared to longer light wavelength or darkness [53]. Higher performance speed to the psychomotor vigilance task (PVT) is also observed when exposed to blue-enriched light exposure as compared to longer-enriched lights [4, 63, 64]. Likewise, blue monochromatic light exposure, as compared to green and red monochromatic lights, induces higher amplitude levels on the P300, an event-related potential associated with attentional demands [65].

Since 2004, a number of studies investigated the brain mechanisms underlying the stimulating effects of light on alertness and cognitive functions in humans [5, 56, 57, 66–72]. These investigations showed that light exposure, particularly blue light, during the execution of cognitive tasks potentiate brain activations of subcortical structures associated with vigilance including the hypothalamus, brainstem (LC), thalamus, and limbic areas (the amygdala and hippocampus) likely before spreading to cortical regions engaged in the ongoing task [5]. Recently and according to a theory of melanopsin bistable properties [59, 73, 74], long wavelength light exposure (589 nm) administered an hour before a given test light exposure increases the impact of that test light on some brain responses (i.e., pulvinar, cerebellum, frontal areas) associated with the execution of a cognitive task [75]. Overall, these studies confirmed that in young subjects, light exposure, particularly blue light, has greater modulating effects on cognitive brain functions than other light wavelengths most likely through melanopsin photoreception and triggers brain activation increases in regions related to alertness and to executive functions (for a review, see [5, 76]).

### Aging and non-image-forming system modifications

Age-related differences in the impact of light have been reported for some acute non-visual responses, with a decreased effect of monochromatic blue light (456 nm) on clock gene expression, subjective alertness, sleepiness, and mood in older, as compared to young individuals [77–79]. However, some investigations did not find age-related reduction in the impact of light when using polychromatic white light [80–82]. A potential decrease in the impact of light remains therefore debated, and it could be that age-related changes occur for specific wavelengths of light or for specific NIF responses but not for others.

Age-related modifications from the eye to the brain may affect the NIF system and contribute to lower sensitivity to light in aging [83–89]. Circadian oscillations are driven by rhythmic expression of clock genes and auto-regulatory transcriptional-translational feedback loops over approximately a 24-h period. Aging appears to be associated with changes in clock gene expression with a reduced amplitude in *Bmal1* and *Clock* expression in SCN [90–92], lower *Per2 expression* in the pituitary gland [93], and lower *Per 1,2,3* expression at the peripheral level (liver, heart) [94]. Age-related differences under light exposure were also revealed including reduction in *Per1* expression after light pulses [90–92] and reduction of *Per2* expression following blue morning light exposure [79]. Since *Per 1–2* expression is rapidly induced by light and is required for entrainment, age-related temporal disorganization may partly result from lower SCN sensitivity to photic stimulation (for a review, see [95]).

Age-related modifications among other molecular and neuronal factors might also contribute to decrease sensitivity to light. Several studies have reported age-related changes in the rhythmic synthesis, release, and expression of vasoactive intestinal polypeptide (VIP) and arginine vasopressine (AVP), two important neuropeptides expressed in the SCN [95–102]. These changes might affect the precision and robustness of rhythmic information transmission by the SCN to other neural sites and might contribute to attenuated photic input signal of the circadian timing system in aging [99, 100, 102, 103]. Other alterations such as a reduction in gray and white matter and changes in vascularization of the brain might contribute to age-related modifications of the NIF system [104–107]. Specifically, decrease density of norepinephrine (NE) neurons in the LC [108], SCN deficits in membrane properties and GABAergic postsynaptic current amplitude [88], and hypertrophy of astrocytes and microglia in SCN (responsible for glutamate uptake—the main neurotransmitter of the RHT) [109] have been reported. Again, all these modifications might induce functional deficits among various systems including the non-image-forming one.

Last but not least, many important age-related changes also occur at the eye level: there is a decrease of photoreceptor sensitivity, a reduction in pupil size, known as senile miosis, and an increase of ocular crystalline lens absorption known as “lens yellowing” [85, 86, 110–115]. The combination of all these changes is very likely to reduce the amount of light reaching the retina and may modulate the impact of light on NIF functions.

### Pupillary constriction and brain sensitivity to light in the course of aging

In order to improve the understanding of the impact of light on non-visual older subjects, we completed two research protocols. We aimed at measuring pupillary constriction [116] and non-visual cognitive brain activity while exposed to light [117]. We recruited two groups of subjects, 16 young and 14 older individuals (see [116] and [117] for complete sample description). All were healthy, right handed, non-smokers, slept between 7 and 9 h per night, were non-medicated, and MRI compatible. They also underwent an optometric exam to make sure they were free of ocular disease. The main hypothesis of our investigations was that in older, compared to young subjects, we would detect a reduction in pupillary and brain responses to light.

#### Pupillary constriction in relation with healthy aging

In the pupillometry protocol, subjects were first maintained in darkness for 15 min before we captured baseline pupil size. Subjects were then exposed for 45 s to three irradiances levels of blue (480 nm) and green (550 nm) monochromatic light (low 7 × 10^12^ ph/cm^2^/s, medium 3 × 10^13^ ph/cm^2^/s, high 1 × 10^14^ ph/cm^2^/s). Resting period in darkness lasted 2 min between each light exposure.

**Fig. 2:**
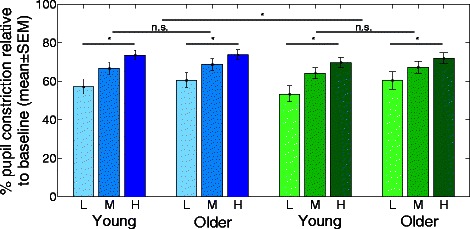
Steady-state pupil constriction in young and older individuals. Mean pupillary constriction relative to baseline ± SEM in each age group. *Blue bars*: blue light at low (*L*), medium (*M*), and high (*H*) irradiances. *Green bars*: green light at low (*L*), medium (*M*), and high (*H*) irradiances. Effects of wavelength and irradiance levels were significant (*p < 0.05), but there was no difference between age groups and no interaction with age (*n.s.* not significant). Reproduced with permission from [116]

As expected, at the baseline (before any light exposure), analysis of the raw pupil size area showed that older subjects have a smaller pupil as compared to young subjects [116]. As PLR was the NIF response of interest, we subsequently estimated the sustained pupillary constriction for each age group under each light condition. Normalized pupillary constriction was calculated for each subject using the value under light exposure in relation with the baseline pupil size. As illustrated in Fig. 2, results showed that pupillary constriction was greater with blue than green light and greater at higher irradiance. However, analysis did not reveal significant age-related differences for sustained pupillary constriction. Our results concur with senile miosis, as absolute pupil size was smaller with age. According to the peak sensitivity of the NIF system, we also observed greater effects of blue rather than green lights and higher rather than lower irradiances. However, similar sustained pupillary constriction was observed in both age groups suggesting that despite a reduction of the amount of light reaching the retina, this non-visual response to light is maintained in healthy aging.

Our first study confirms the reduction in pupil size with aging and the greater impact of blue versus. green light on PLR [116] but does not reveal significant age-related differences in pupil dynamic under light exposure. This original result indicates that PLR might differ from other acute non-visual responses showing a decrease in sensitivity to blue light with age (i.e., suppression of melatonin secretion, modulation subjective alertness, mood and the expression of certain clock genes) [77–79].

As previously exposed, different NIF responses are regulated by partially independent neural networks [23, 30, 118–120]. These anatomical differences support the possibility of variations in the age-related changes in effects of light on various NIF functions, sustained for instance by the OPN (PLR) or the SCN (entrainment). Specific light sensitivities for different NIF responses [121, 122] might also contribute to the diversity in the changes in the impact of light in aging. Animal evidences revealed indeed higher sensitivity thresholds (i.e., requiring higher light level) for the circadian entrainment phase response and masking (i.e., motor activity suppression in nocturnal animals under light exposure) than for pupillary constriction [121, 122]. It is plausible that the sensitivity threshold of the pupillary reflex is low enough to trigger a pupillary response similar to that of young people despite the reduction of photic input reaching the retina.

#### Brain sensitivity to light, cognition, and healthy aging

For the neuroimaging study, the same two groups of subjects completed an fMRI recording at night, 1 h after their habitual sleep time. They had to follow a regular sleep schedule 7 days prior to the experiment and were maintained in darkness 2 h before the experimental light exposure. In the scanner, subjects completed 28 blocks of 45 s of the auditory working memory two-back task while maintained in a darkness condition or under blue monochromatic light of three irradiance levels (low 7 × 10^12^ ph/cm^2^/s, medium 3 × 10^13^ ph/cm^2^/s, high 1 × 10^14^ ph/cm^2^/s). The two-back task required the subjects to answer, with a response box, whether each letter presented was the same as the two prior letters. This task engaged auditory processing, attention, storing, comparing, and updating information in working memory [123]. Subjects were well trained to the task prior to the fMRI recordings. Consequently, behavioral analyses revealed no significant differences between the two groups and between the four light conditions for accuracy and response time values [117]. This was intended and consistent with a ceiling effect in both groups, so that the limited amount of light we administered could not significantly impact performance. This situation was ideal for the purpose of our study which was to investigate the brain mechanisms involved in the impact of light as we are sure that behavior did not significantly bias our fMRI results.

**Fig. 3:**
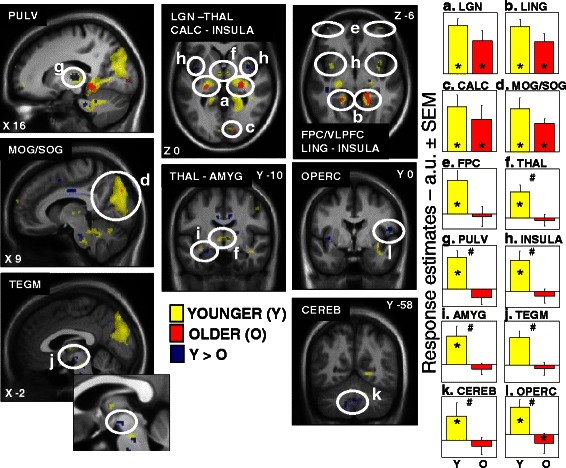
Effect of the presence of light on brain responses of younger and older individuals performing an auditory two-back task. Statistical results (*p* < 0.001 uncorrected) overlaid over the mean structural image of all participants. Significant responses to light are displayed in yellow for younger individuals (*Y*), in red for older individuals (*O*), whereas group differences (*Y* > *O*) are in *blue. Right* panels **a-l** show activity estimates (arbitrary unit (a.u.) ± standard error of the mean) in each brain region. *Significantly activated, *p* < 0.05 corrected for multiple comparisons over small volumes of interest; *#* significant group differences, *p* < 0.05 corrected for multiple comparisons over small volumes of interest. Abbreviations: **a** *LGN* lateral geniculate nucleus, **b** *LING* lingual gyrus, **c** *CALC* calcarine sulcus, **d** *MOG/SOG* middle and superior occipital gyrus, **e** *FPC* frontopolar cortex, **f** *THAL* dorsomedian thalamus, **g** *PULV* thalamus pulvinar, **h** *INSULA* insula, **i** *AMYG* amygdala, **j** *TEGM* tegmentum, **k** *CEREB* cerebellum, **l** *OPERC* frontal operculum. Please refer to Table 2 of [113] for brain clusters coordinates. Reproduced with permission from [117]

In accordance with literature, and supporting that the subjects performed the task correctly, we first showed brain activations in areas known to be involved in the task including the frontal gyrus, the superior parietal and temporal gyrus, the intraparietal sulcus (IPS), the motor and sensorimotor cortices as well as the thalamus, and the cerebellum [117]. We also investigated which brain areas responded to the presence of light during the execution of the task, independently of the irradiance levels, in young and older subjects. Results indicated common brain activations in young and older individuals in the LGN, the lingual gyrus, the calcarine sulcus, and in the occipital gyrus. These common brain activations in relation with the effects of light are presented in Fig. 3.

Analysis also revealed significant age-related differences as young subjects presented a higher impact of light than older subjects (represented in blue in Fig. 3) in the thalamus and a region compatible with the ventral tegmental area (VTA), important areas for arousal regulation [124], in the amygdala and the insular cortex, regions involved in emotional regulation [125], as well as in the frontal operculum and in the cerebellum. Some of these regions have been previously reported in non-visual effects of light in young subjects and are part of the salience brain network engaged in the selection of most relevant information to guide behavior [45, 126]. Less brain sensitivity to light among regions of this network might have important impacts on brain sensitivity to light in aging on alertness and attention.

**Fig. 4:**
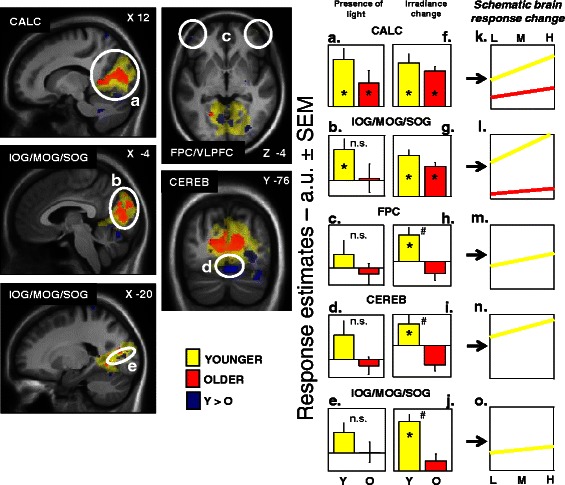
Effect of irradiance level of light on brain responses of younger and older individuals performing an auditory two-back task. Statistical results (*p* < 0.001 uncorrected) overlaid over the mean structural image of all participants. Significant changes in responses as a function of irradiance level are displayed in *yellow* for younger individuals (*Y*) and in *red* for older individuals (*O*), whereas group differences (*Y* > *O*) are in *blue*. Panels **a–e** represent estimates [a.u. ± standard error of the mean (SEM)] of the brain responses while exposed to blue light independent of the irradiance change. Panels **f–j** represent estimates (a.u. ± SEM) of the linear change in brain responses with change in irradiance level. Panels **k–o** consist of a schematic representation of the composite of both components (responses to light and irradiance change) showing the evolution of the responses with change in irradiance level. Panels **m–o** only includes younger  individuals because responses were not significantly affected by irradiance change in older individuals in the brain regions considered. *Significantly activated, *p* < 0.05 corrected for multiple comparisons over small volumes of interest; ^*#*^significant group differences, *p* < 0.05 corrected for multiple comparisons over small volumes of interest; *n.s.* non significant group difference. Abbreviations: **a** & **f** *CALC* calcarine sulcus, **b**, **e**, **g**, & **j** *IOG/MOG/SOG* inferior/middle/superior occipital gyrus, **c** & **h** *FPC* frontopolar cortex, **d** & **i** *CEREB* cerebellum. Please refer to Table 3 of [113] for brain clusters coordinates. Reproduced with permission from [117]

We also investigated which brain areas responded differently with age to increasing blue light irradiance levels during the ongoing cognitive task. Again, results showed common brain activations in young and older subjects in the calcarine sulcus, as well as in the inferior, median, and superior occipital gyrus. As represented in Fig. 4, these regions seem to increase their activation with increased light intensity in both groups. More importantly, our results also pointed toward age-related differences in the prefrontal cortex, an important region for higher cognitive functions [127], in the occipital cortex, a region related to the visual system, and finally, in the cerebellum. Our results suggested an increase in frontal, occipital, and cerebellum brain activations in young subjects following light increase intensity, while in older subjects, this phenomenon was absent.

Overall, these results indicated that light is still able to modify ongoing brain activity in older individuals in the context of our protocol. Age-related modifications are also evident at the irradiance levels we used. Based on our results, one could argue that light impact is better conserved in aging in brain areas that are typically associated with vision (LGN, calcarine sulcus, and occipital areas), while areas involved in alertness and cognition regulation seem to undergo a more pronounced diminution in their response to light.

Reduced age-related effects of blue monochromatic light on the thalamus and VTA activity might be related to various molecular and neural changes in the arousal system. Hypocretin/orexin neurons, the expression of which decreases with age [128], innervate many cell groups including “wake-active” monoaminergic populations of the VTA [129–132]. A reduced impact of blue monochromatic light in the VTA-compatible area suggests that the dopaminergic system could be involved in age-related changes of the stimulating effect of light on brain responses. The VTA is an important source of dopamine in the brain and is crucial both for the regulation of sleep and alertness and for cognition and mood [124]. It is notable that the VTA sends projections to the SCN [133]. Since dopamine dysfunction is thought to play an important role in the cognitive decline found in healthy aging [134], the reduced effect of light upon brain-related dopamine regions might contribute to reduce the stimulating effect of blue light on cognitive functions.

## Conclusions

### Lighting-up the aging brain

Light is a simple mean that could easily be used to improve cognition, sleepiness, mood, and sleep in normal and pathological aging. Daytime sleepiness is a significant characteristic of specific neurodegenerative disorders and is associated with not only current cognitive impairments but also increased risks for developing cognitive decline [135–141]. In Alzheimer’s and Parkinson’s disease patients, excessive sleepiness and fatigue have been associated with increased functional impairment [142] and cognitive dysfunction [143]. While Parkinson’s disease is directly related to dopamine dysfunction [144], a slow degeneration of hypocretin neurons has been reported over the course of Alzheimer’s disease [130]. Importantly, light exposure has a positive effect on sleep and mood in Parkinson’s disease patients and improvement of cognitive functions have been reported using 2 h of bright light therapy (polychromatic light—3000 lx and over) in Alzheimer’s disease patients [145, 146]. Qualitative positive effects of light exposure on sleep, mood, and cognition have also been reported in Alzheimer’s disease patients with greater effect of blue-green bright light exposure in the morning as compared to dim red light [147].

The spectral quality of light may be a crucial factor to consider when dealing with light in aging. Besides monochromatic light, one could use polychromatic light, enriched in blue wavelength for instance. These would be more applicable to real life and have been reported to improve some aspects of cognitive performance relative to classical incandescent light [64, 148]. Each non-visual response to light will require special attention as they may be differently affected by age since they rely in part on different photoreceptor contributions and partly independent brain pathways.

Furthermore, investigations need to identify light characteristics (quality, quantity, duration) that can effectively modulate alertness and cognitive performance in aging. In order to reach a better understanding of the eye factors upon brain sensitivity to light, future investigations need to measure pupil size at the time of the experience or to include older subjects who underwent lens replacement following a cataract surgery. As it is now recognized that melanopsin gene polymorphism (OPN4) influences pupil size under light exposure [149, 150] and that clock gene polymorphism (PER3) influences non-visual sensitivity to light according to sleep pressure and circadian phase [57, 63, 75], it is also crucial to consider genetics—age interactions. Aside from pharmacology, we may then be in a position to provide light tools to improve life quality in aging.

### Ethics approval and consent to participate

Our experiments received Institutional ethics approval from the Research ethics board of the *Comité mixte d'éthique de la recherche du Regroupement Neuroimagerie Québec (CMER-RNQ) * and written informed consent was obtained from each participant.
